# Tumor-Originated Exosomal hsa-miR-3937 as a Minimally Invasive Early Biomarker for Liquid Biopsy of Colorectal Cancer

**DOI:** 10.1155/2022/6990955

**Published:** 2022-05-11

**Authors:** Dan Qiao, Chenzheng Gu, Weiwei Wang, Wenhui Yan, Chenfei Jiang, Jingwen Hu, Anquan Shang, Jian Guo

**Affiliations:** ^1^Department of Laboratory Medicine, Ruijin Hospital, Shanghai Jiao Tong University School of Medicine, Shanghai 200025, China; ^2^Department of Laboratory Medicine, Shanghai Tongji Hospital, School of Medicine, Tongji University, Shanghai 200065, China; ^3^Department of Pathology, Tinghu People's Hospital of Yancheng City, Yancheng 224001, China; ^4^Department of Laboratory Medicine, Yangzhi Rehabilitation Hospital (Shanghai Sunshine Rehabilitation Center), Tongji University School of Medicine, No. 2209 Guangxing Rd, Shanghai 201619, China; ^5^The College of Medical Technology, Shanghai University of Medicine & Health Sciences, Shanghai 200237, China; ^6^Department of Laboratory Medicine, Shanghai East Hospital, School of Medicine, Tongji University, Shanghai 200120, China

## Abstract

**Background:**

Exosomal microRNAs (miRNAs) have been linked to the genesis and progression of certain cancers. The role and regulation mechanism of cancer-derived exosomal miRNAs in CRC, however, remain unknown.

**Methods:**

To address this, we first used miRNA sequencing to describe the miRNA profiles of circulating exosomes in order to identify miRNAs that were differently expressed between patients with CRC and healthy controls. Transmission electron microscopy, nanoparticle tracking analysis (NTA), and western blot were used to analyze exosomes generated from CRC cells. CCK-8, wound healing, and Transwell tests were used to see whether exosomes affected CRC cell proliferation, metastasis, and apoptosis, respectively. We chose and identified hsa-miR-3937, which was abundant in tumor-generated exosomes, based on earlier RNA sequencing data of exosomes obtained and extracted from seven matched specimens of tumor tissues and surrounding normal tissues of CRC patients.

**Results:**

The role of hsa-miR-3937 in CRC cells was found, and silencing of hsa-miR-3937 decreased CRC cell invasion and migration in a Transwell experiment. Furthermore, we discovered that there was no link between hsa-miR-3937 expression and CRC cell apoptosis. Initially, it was discovered that BCL2L12 was the target gene of hsa-miR-3937, and the TCGA database highlighted the potential therapeutic relevance of BCL2L12. Furthermore, to identify hsa-miR-3937 as a biomarker of CRC, we used peripheral blood samples rather than patient tissues and extracted exosomes from plasma samples. To assess the expression levels and predictive usefulness of plasma exosomal hsa-miR-3937 in CRC, we performed RT-qPCR to identify hsa-miR-3937 levels in all samples. We also gathered clinicopathological information in order to look for links between aberrant hsa-miR-3937 expression and clinicopathological characteristics. The pathologic stage of CRC patients was linked to hsa-miR-3937 expression levels, and the same was true for the T stage. ROC curve study revealed that hsa-miR-3937 outperforms CEA and CA199. The combination of hsa-miR-3937, CEA, and CA199 exhibited the highest sensitivity for CRC diagnosis.

**Conclusions:**

Our findings show that the tumor-originated exosomal hsa-miR-3937 is a potential and effective liquid biopsy marker for colorectal cancer detection and therapy.

## 1. Introduction

With more than 1.85 million diagnoses and 850 000 deaths yearly, colorectal cancer (CRC) is the third most prevalent cause of cancer deaths globally [1]. Despite advances in screening and the development of targeted therapies, roughly half of CRC patients still die because of delayed discovery of advanced disease with localized or distant metastases. Many nations have seen an increase in the incidence of early-onset colorectal cancer (usually defined as CRC diagnosed before the age of 50) [2]. Screening for colorectal cancer and precancerous lesions includes stool tests, carcinoembryonic antigen (CEA), colonoscopy, colonography, computed tomography (CT), and double-contrast barium enema (DCBE) [3]. There are a few drawbacks to these approaches, such as the fact that some of them are intrusive or lack sensitivity and specificity. A great need exists to find biomarkers for early identification of CRC and to investigate new therapy targets for CRC.

MicroRNAs (miRNAs) are small noncoding RNAs 18-25 nucleotides in length that downregulate gene expression during various crucial cell processes such as apoptosis, differentiation, and development [4, 5]. The expression of miRNAs has been altered in several human cancers, including colon cancer (CC). miRNAs have been shown to be tumor suppressors and oncogenes, according to functional investigations. CRC alters and appears to be affected by miRNA regulation in many proteins involved in key signaling pathways, such as members of the Wnt/-catenin and phosphatidylinositol-3-kinase (PI3K) pathways, KRAS, p53, extracellular matrix regulators, and transcription factors of the epithelial-mesenchymal transition [6–9]. These results greatly expand Vogelstein's concept of CRC etiology and demonstrate the immense potential of miRNAs as a new class of therapeutic targets. Many studies have shown that miRNA expression patterns may predict CRC prognosis and treatment response, as well as help the identification of CRC among cancers of uncertain source location. Hundreds of miRNA expression profiles have been demonstrated to offer at least the same potential for identifying biomarkers as profiling their mRNA or protein equivalents. The presence of miRNAs in serum and plasma has been consistently documented, and the use of miRNAs as minimally invasive biomarkers for the identification of colon cancer has shown promising results [10].

Exosomes are small extracellular vesicles that are secreted by all cell types in the human body [11, 12]. There is intercellular communication via them. Exosomes are single-membrane organelles with a diameter of 30 to 200 nm that are identical to the cell's structure [13]. RNA, proteins, and lipids are all present in the genomes of organisms. It is possible to use exosomes as a diagnostic for cancer screening since cancer cells produce more exosomes than normal cells. miRNA may be transferred from one cell to another through exosomes, which act as messengers. Target cells are reprogrammed via this communication with regards to invasiveness (gene expression), tumor development (angiogenesis), and immunological function. Exosome-derived exosomes from CRC cells have been shown to include miR-183-5p, which has been shown to boost the proliferation, migration, and tube-forming properties of HMEC-1 cells [14]. Exosome-contained oncogenic microRNAs (miRNAs) regulate EMT as well. To maintain EMT in lung cancer cells, exosomes control the expression of the E-cadherin marker E-cadherin through TGF-*β* [15]. Exosomes have been implicated in an increasing number of research in recent years as a potential cause of medication resistance. Tumor-resistant MCF-7 cells release miR-221/222, which may render sensitive MCF-7 cells resistant to tamoxifen by suppressing p27 and E2A gene expression in sensitive cells (ER*ɑ*) [16]. Researchers have also discovered the diagnostic and prognostic use of exosomal miRNA as a cancer diagnostic and prognostic marker. Exosomal miR-203 was shown to be associated with a greater incidence of liver metastasis and a worse survival rate in patients with advanced colorectal cancer [17]. Advanced TNM staging, lymph node and liver metastases, and poor survival all correlate with higher levels of exomiR-19a and miR-6803-5p [18, 19]. Exosomal miRNAs have been shown to be associated with a worse survival rate in previous research.

Based on these findings, exosomal hsa-miR-3937 has been shown to have a function in CRC, and the study's major goal was to uncover its possible roles and assess its diagnostic and prognostic value in CRC and its effectiveness.

## 2. Materials and Methods

### 2.1. Patients and Specimens

Seven paired specimens of CRC tissues and adjacent normal tissues of CRC patients who underwent surgical resection were provided by Tongji Hospital of Tongji University. And we collected peripheral blood samples from CRC patients and healthy donors from November 2019 to July 2021. None of CRC patients had received radiotherapy or chemotherapy prior to surgery. Written informed consent was obtained from all patients. The study was approved by the Ethics Committee of Shanghai Tongji hospital (2021KYSB061), and the methods were designed according to the Declaration of Helsinki.

### 2.2. Tissue Exosome Isolation

Millimeter-sized fresh tumor and peritumoral adjacent tissue were harvested from patients with localized CRC undergoing resection. The tissue was placed in ice-cold PBS within minutes of collection and submitted for downstream processing and analysis [20]. According to the reagent instructions, commercial tissue dissociation kit (Jiangxi Boling, China) was used for intestinal tissue dissociation. Firstly, the tissue samples were mixed with the preprepared tissue dissociation solution at a ratio of 2 g : 5 ml, and the mixture was placed in a 37°C constant temperature shaker (90 min, 200 rpm). After the dissociation, the suspension was filtered with a 70 *μ*m filter, and the filtrate was collected for centrifugation (3000 g, 4°C, 30 min). The supernatant was centrifuged in a new test tube (13000 g, 4°C, 10 min) and filtered by a 0.22*μ*m filter, and the supernatant was collected. The filtrate of the previous step was centrifuged again at high speed (13000 g, 4°C, 30 min); then, the supernatant was filtered by a 0.22*μ*m filter and transferred to an overspeed centrifuge tube for overspeed centrifugation (100000 g, 4°C, 150 min). After superseparation, the bottom of the tube precipitates are tissue exosomes.

### 2.3. Plasma Exosome Isolation

Plasma exosomes were extracted by BestBio-Exosome kit (BestBio, China) under the instruction. Firstly, we isolated the plasma of peripheral blood at room temperature. Then samples were centrifuged at 3000 g for 15 minutes to remove residual cells and cell debris [21]. Next, samples were centrifuged at 10000 g for 20 minutes to remove microvesicles. 500 *μ*l supernatant was removed into a new tube with 500 *μ*l A reagent and 250 B reagent of kit. This new tube was thoroughly mixed and incubated at 4°C at least 10 hours. After these steps, samples were centrifuged at 10000 g for 60 minutes. Finally, remove the supernatant of tubes and resuspend the sediments with 1x PBS, and the remaining pellet was deemed as the exosome fraction.

### 2.4. Transmission Electron Microscopy (TEM)

Transmission electron microscopy was performed as previously described [22]. The following primary materials and instruments were used for TEM: 1x PBS (Sigma, USA), 1% glutaraldehyde (Solarbio, China), 2% uranyl-oxalate solution at pH 7, 2% (Solarbio, China), methylcellulose at pH 4 (Solarbio, China), 4% uranyl acetate (Solarbio, China), and the FEI TecnaiG2 spirit transmission electron microscope (Thermo-Fischer, Waltham, USA).

### 2.5. Nanosight Particle Tracking Analysis (NTA)

Nanosight particle tracking analysis was performed as previously described [22]. Nanoparticle tracking analysis (NTA) (NanoSight NS300, Malvern Instruments, UK) was used for exosomes size distribution and concentration.

### 2.6. Western Blot Analysis

Cells or exosomes were isolated and denatured in sodium dodecyl sulfate (SDS) buffer for western blot analysis. Primary antibodies of exosome markers such as negative marker: GM130 and positive marker: TSG101 were obtained from Cell Signaling Technology (CST, USA). Secondary antibodies were goat-anti-rabbit from Dako (Carpinteria, USA).

### 2.7. RNA Sequencing and Data Analysis

Exosomes were isolated from CRC tissues and adjacent normal tissues of CRC patients. Total RNAs in exosomes were extracted using the Total Exosome RNA and Protein Isolation Kit (Invitrogen, USA) as the manufacturer's protocol. The amount and quality of small RNA in the total RNA were tested by Ribobio Co. Ltd. Small RNA library construction and sequencing were performed by Ribobio Co. Ltd. Then, the cDNA library was sequenced on Illumina HiSeq 2500. Raw reads were collected using the Illumina analysis software.

### 2.8. Cell Line Culture

Human CRC cell lines HCT116 and SW480 were obtained from American Type Culture Collection. HCT116 and SW480 cells lines were cultured in DMEM medium (Hyclone, USA) supplemented with 10% fetal bovine serum (FBS) (Sigma, USA) and 1% penicillin-streptomycin (Invitrogen, USA) in a 37°C humidified incubator with 95% air and 5% CO_2_.

### 2.9. Transfection and Plasmid Construction

The hsa-miR-3937 mimic, inhibitor, siRNA-BCL2L12, and a respective negative control (Obio, China) were transfected into cells in 6-well plates using Lipofectamine™ 2000 (Invitrogen, USA) when the cells were approximately 70% confluent, culturing in medium without FBS for 9 hours. Then, the medium was replaced by that supplemented with 10% FBS. The cells were harvested 48 hours after transfection, and the effect of transfection was assessed by real-time quantitative polymerase chain reaction (qRT-PCR).

### 2.10. RNA Extraction and qRT-PCR

Total RNA was extracted from the transfected cells and exosomes using TRIzol reagent (Qiagen, USA). To quantify hsa-miR-3937, all-in-one miRNA qRT-PCR kits (GeneCopoeia, USA) were utilized with hsa-miR-3937-specific primers (Ribobio, China) according to the manufacturers' instructions. To quantify BCL2L12, cDNA was reverse transcribed using PrimeScript RT reagent kit (Takara, China). qRT-PCR was performed by using SYBR Green Master Mix (Accurate Biology, China). We choose U6 as internal control of miRNA and GAPDH as internal control of mRNA.

The primer sequences were as follows: BCL2L12 (forward): 5′-CCTGTTCCAACTCCACCTAGAA-3′; BCL2L12 (reverse): 5′-GACTCAGAGGGGGCTGCT-3′; GAPDH (forward): 5′-GTCTTCACCACCATGGAGAA-3′; and GAPDH (reverse): 5′-TAAGCAGTTGGTGGTGCAG-3′. All the experiments were performed in triplicate on an ABI7500 Sequence Detection System, and the mean cycle threshold (CT) data were obtained. The relative amount normalized to the internal control was calculated with the equation 2^-*ΔΔ*CT^.

### 2.11. Cell Apoptosis Assay

The transfected cells were stained using the TUNEL Assay kit (Beyotime, China) in accordance with the manufacturer's steps. In addition, we used the Annexin V-FITC/PI Apoptosis Detection Kit (BD, USA) to analyze the apoptosis of cells through a flow cytometry, and the percentage of the apoptosis cells was quantified using FlowJo software.

### 2.12. Cell Proliferation Assay

The transfected cells were plated at 2 × 10^3^ cells/well in 96-well plates and incubated overnight in medium supplemented with 10% FBS. Cell proliferation was measured using a Cell Counting Kit-8 (Beyotime, China) at 24, 48, and 72 hours under the instructions of protocols. The absorbance at 450 nm was measured using microplate readers (Synergy H4 Hybrid Reader, BioTek, USA). The data are representative of three independent experiments in triplicate.

### 2.13. Transwell Migration and Invasion Assay

Migration and invasion assay were performed as previously described [22]. The materials and instruments included Transwell chambers (Corning, USA), 5% crystal violet (Beyotime, China), and Nikon Inverted Research Microscope Eclipse Ti microscope.

### 2.14. Luciferase Reporter Assay

Two sets of luciferase reporter plasmids were constructed, and each set of plasmids contained a fragment of BCL2L12 3′-untranslational region (3′-UTR) with a conserved hsa-miR-3937 binding site (Obio, China). For the luciferase reporter assays, 4 × 10^4^ CRC cells were seeded into a 24-well plate the day before transfection, and each well was cotransfected with firefly luciferase reporter plasmids, hsa-miR-3937 mimic, or the negative control using Lipofectamine™ 2000 (Invitrogen, USA). After transfection for 48 hours, the luciferase activities were evaluated using the Dual Luciferase Reporter Assay Kit (Promega, USA) following the manufacturer's instructions.

### 2.15. Database's Analysis

We utilized the online database TargetScan to predicted target genes of hsa-miR-3937 and confirmed the specific binding sites for hsa-miR-3937 on the 3′-UTR region of the target gene. Additionally, the online database (https://cm.jefferson.edu/rna22/Interactive/) was used to elucidate the function of hsa-miR-3937 and its target gene BCL2L12.

### 2.16. Statistical Analysis

The results of data are expressed as mean ± s.d. of three independent biological experiments. Student' s *t*-test was performed to analyze two groups, and one-way analysis of variance (ANOVA) was used to calculate multiple groups. Rx64 3.6.1 was conducted to process data, analyze data, and plot diagrams in this study. Differences were considered statistically significant at *p* value < 0.05.

## 3. Results

### 3.1. Exosomes Secreted from CRC Tissues Exhibit High hsa-miR-3937 Expression

**Figure 1:**
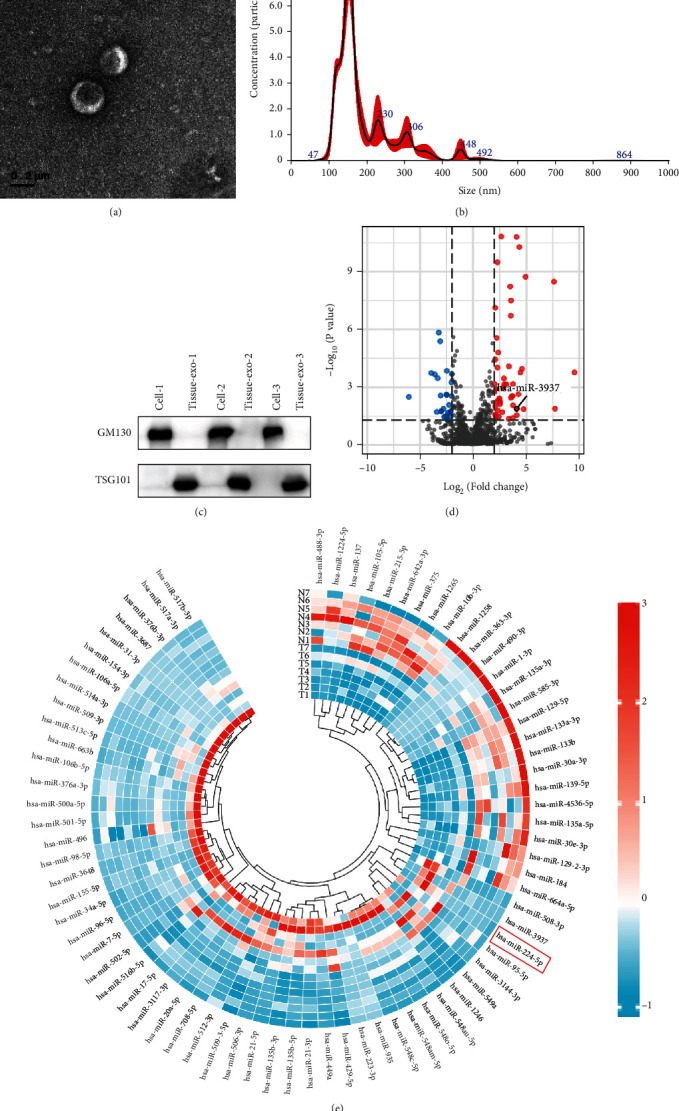
hsa-miR-3937 is significantly upregulated in CRC tumor-derived exosomes. (a) Exosomes were isolated from CRC tissues and adjacent normal tissues, and the characteristics were identified by TEM. (b) NTA distribution of CRC tumor-derived exosomes. (c) Western blot was utilized to identify markers of exosomes. (d) Volcano plot of the expression profile by RNA sequencing. (e) Heatmap of the expression profiles.

miRNAs have been shown to be overrepresented in tumor-derived exosomes. To discover CRC-related miRNAs, we examined the expression profile in exosomes from seven paired CRC tissues and adjacent normal tissues from CRC patients who had surgical resection at Tongji Hospital of Tongji University. Firstly, we collected and identified the tissue-derived exosomes. These exosomes were detected by TEM and NTA methods and were found as rounded particles with approximately 150 nm in size with a double-layer membrane (Figures 1(a) and 1(b)). These characteristics were consistent with common size of exosomes in reviews. The tissue-derived exosomes were also characterized by western blot analysis with the introduced expressions of exosome-specific markers, including a negative marker: GM130 and a positive marker: TSG101 (Figure 1(c)). Then, we analyzed the RNA sequencing results, which showed that has-miR-3937 was significantly upregulated in CRC tissue-derived exosomes (Figure 1(d)).

### 3.2. Prognostic Value of hsa-miR-3937 in CRC by TCGA

**Figure 2:**
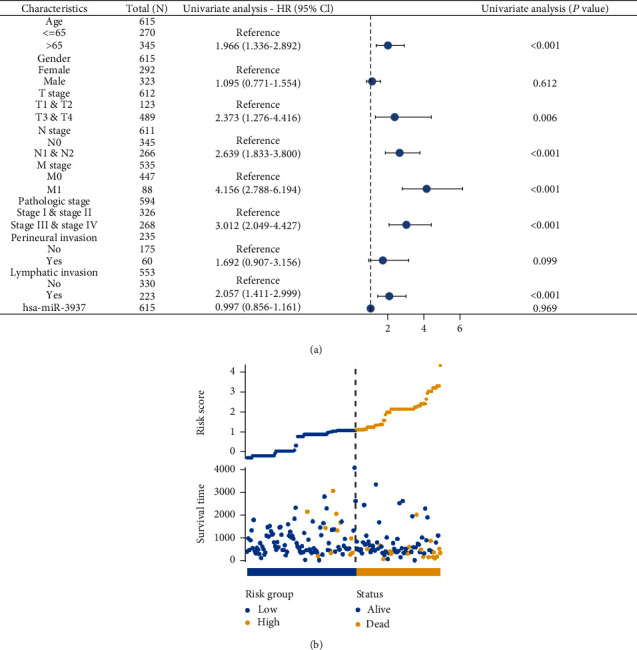
Prognostic value of hsa-miR-3937 in CRC by TCGA. (a) Univariate analysis of hsa-miR-3937 in CRC by TCGA. (b) hsa-miR-3937 as a risk factor to predict prognostic value in CRC (ns, *p* > 0.05; ∗, *p* < 0.05; ∗∗, *p* < 0.01; ∗∗∗, *p* < 0.001; ∗∗∗∗, *p* < 0.0001).

To further uncover the impact of patients' has-miR-3937 expression in CRC, we used the univariate Cox regression analysis to assess the correlation between has-miR-3937 expression and overall survival in two cohorts. The has-miR-3937 was then tested as an independent predictive factor for CRC patients' survival. The Cox regression analysis of has-miR-3937 has statistical significance when *p* < 0.05. In univariate analysis, hsa-miR-3937 was an independent factor of age, T stage, N stage, M stage, pathologic stage, and lymphatic invasion (Figure 2(a)). In addition, we evaluated the risk score of hsa-miR-3937 in CRC patients and revealed that the high-risk group had a higher mortality rate (Figure 2(b)). These results showed that there was further clinical value of hsa-miR-3937 in CRC patients.

### 3.3. Overexpression of hsa-miR-3937 Promoted CRC Cell Proliferation and Silencing of hsa-miR-3937 Inhibited CRC Cell Invasion and Migration

**Figure 3:**
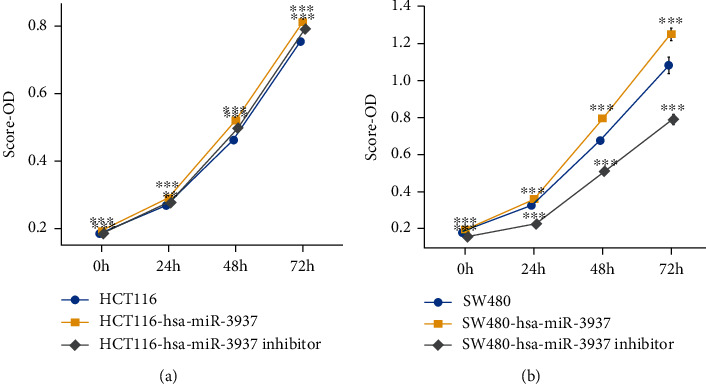
Overexpression of hsa-miR-3937 promoted CRC cell proliferation. (a and b) Overexpression of hsa-miR-3937 promoted HCT116 and SW480 cell proliferation (ns, *p* > 0.05; ∗, *p* < 0.05; ∗∗, *p* < 0.01; ∗∗∗, *p* < 0.001; ∗∗∗∗, *p* < 0.0001).

**Figure 4:**
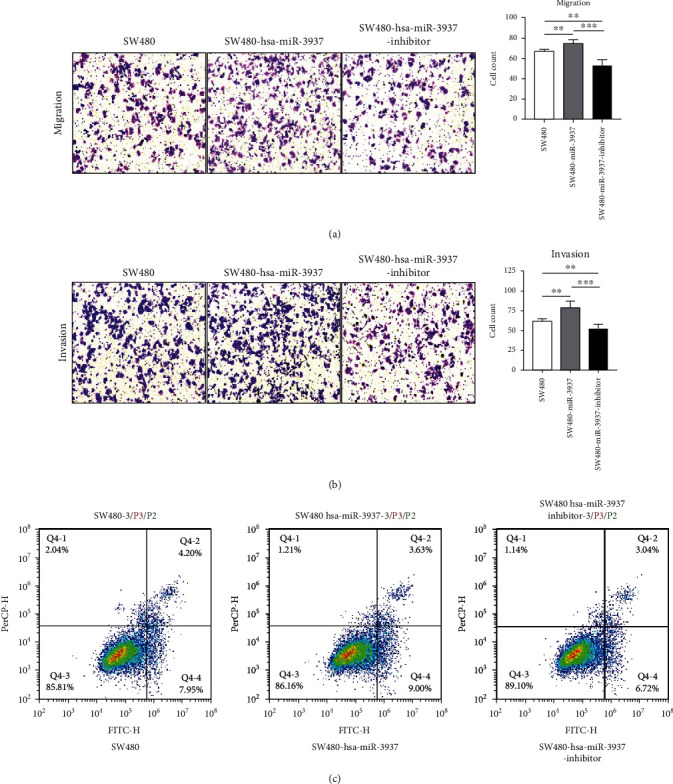
Silencing of hsa-miR-3937 inhibited CRC cell invasion and migration. (a and b) SW480 migration and invasion assays using Transwell with or without Matrigel. (c) There was no obvious association between hsa-miR-3937 expression and cell apoptosis (∗∗, *p* < 0.01; ∗∗∗, *p* < 0.001).

To determine whether hsa-miR-3937 modulated CRC tumorigenesis, HCT116 and SW480 cells with a certain hsa-miR-3937 expression were transfected with hsa-miR-3937 mimic, inhibitor, and their negative controls. The expression level was confirmed by qRT-PCR. As the evident from CCK-8 assay shown, overexpression of hsa-miR-3937 induced the viability of CRC cells, with the results that overexpression of hsa-miR-3937 cells had significantly higher proliferation (Figures 3(a) and 3(b)). Moreover, Transwell assay showed that inhibition of hsa-miR-3937 resulted in a significant decrease in the number of migrating (Figure 4(a)) and invading cells compared with control cells (Figure 4(b)). In addition, TUNEL assay in SW480 cell line revealed that there was no significant difference between the expression level of hsa-miR-3937 and apoptotic rate (Figure 4(a)). In addition, flow cytometry revealed that there was no significant difference between the expression level of hsa-miR-3937 and apoptosis (Figure 4(c)). The apoptosis assay may indicate that the migration and invasion of hsa-miR-3937 in CRC tumorigenesis was not associated with the apoptosis of CRC cell but another downstream molecular function.

### 3.4. BCL2L12 Was a Direct Target of hsa-miR-3937

**Figure 5:**
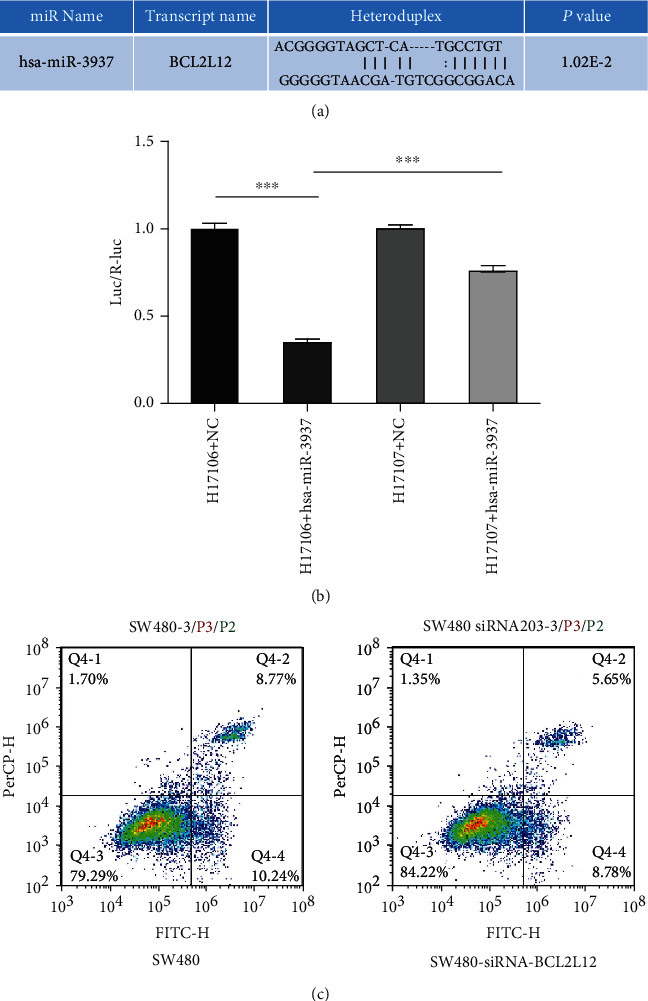
BCL2L12 was a target gene of hsa-miR-3937. (a) TargetScan was used to predicted the binding site of hsa-miR-3937 on the target gene BCL2L12. (b) Luciferase reporter was performed to examine the binding ability between hsa-miR-3937 and BCL2L12. (c) Cell apoptosis was used to analyze the function of SW480 after interfering BCL2L12 expression (ns, *p* > 0.05; ∗, *p* < 0.05; ∗∗, *p* < 0.01; ∗∗∗, *p* < 0.001; ∗∗∗∗, *p* < 0.0001).

To gain insight into the mechanism by which hsa-miR-3937 acts as an oncogenic miRNA in CRC cells, we performed a prediction based on TargetScan, displaying the binding site of hsa-miR-3937 on the target gene BCL2L12 (Figure 5(a)). A luciferase reporter assay was performed to verify whether BCL2L12 was indeed the target of hsa-miR-3937. We build two new plasmids of BCL2L12 which were pMIR-REPORT Luciferase-BCL2L12 3′-UTR (WT) H17106 and pMIR-REPORT Luciferase-BCL2L12 3′-UTR (MUT) H17107. The results indicated that hsa-miR-3937 reduced the luciferase activity of BCL2L12-WT (*p* < 0.001), while this was not obviously observed in the luciferase activity of BCL2L12-MUT (*p* > 0 05), indicating that hsa-miR-3937 could bind to and downregulate the expression of BCL2L12 (Figure 5(b)). And in SW480 cell line, downregulating of BCL2L12 could inhibit the apoptotic rate of cells, with the results that the number of siRNA-BCL2L12 (14.43%) was lower than that in control group (19.01%) (Figure 5(c)).

### 3.5. The Potential Function of BCL2L12 in CRC by TCGA

**Figure 6:**
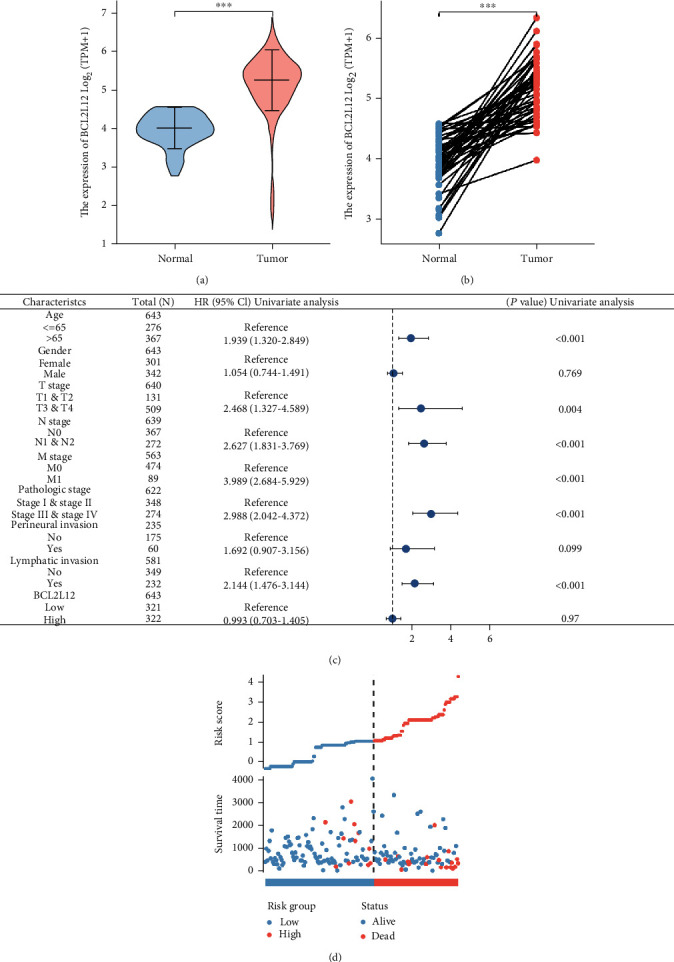
The potential function of BCL2L12 in CRC by TCGA. (a and b) BCL2L12 mRNA expression level was higher in CRC tissues than normal tissues. (c) Univariate analysis of BCL2L12 in CRC by TCGA. (d) BCL2L12 as a risk factor to predict prognostic value in CRC (ns, *p* > 0.05; ∗, *p* < 0.05; ∗∗, *p* < 0.01; ∗∗∗, *p* < 0.001; ∗∗∗∗, *p* < 0.0001).

**Figure 7:**
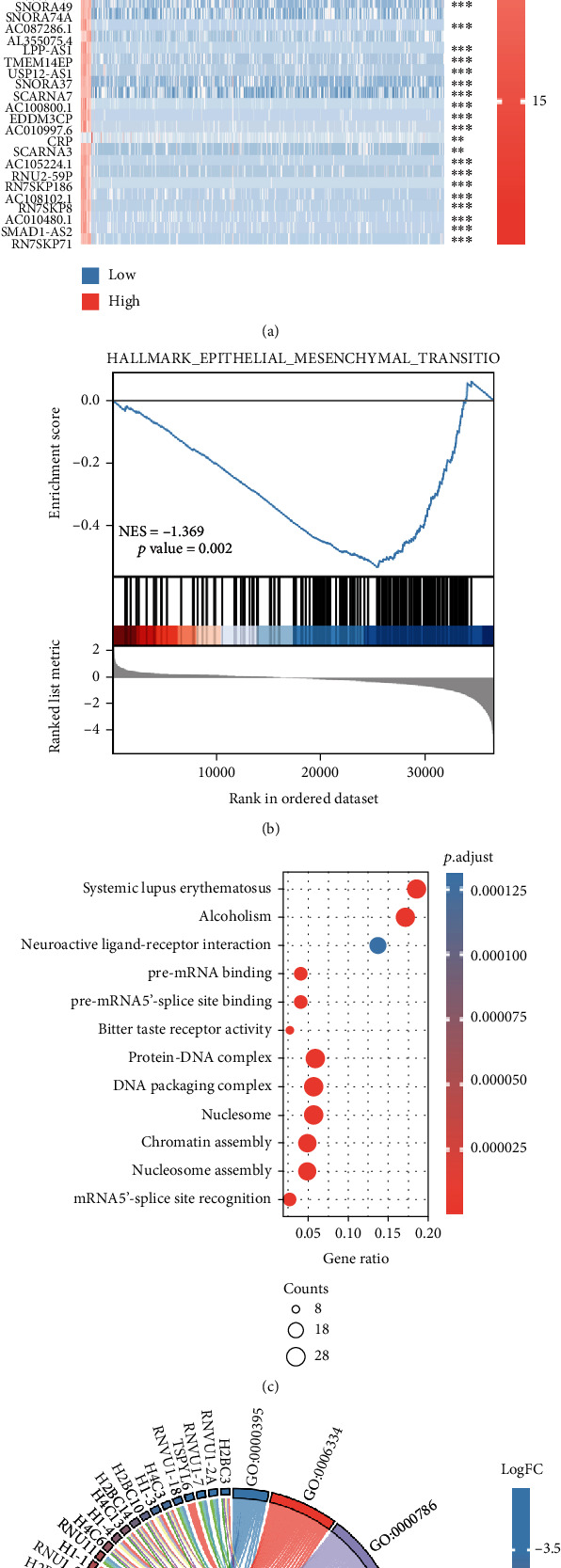
Differential expression genes of BCL2L12 in CRC and potential downstream pathways by TCGA. (a) The heatmap showed the top 50 of DEGs related with BCL2L12 in CRC. (b) GSEA results of DEGs associated with BCL2L12 in CRC by TCGA database, enriching in EMT signature. (c and d) KEGG and GO to predict the downstream pathways (ns, *p* > 0.05; ∗, *p* < 0.05; ∗∗, *p* < 0.01; ∗∗∗, *p* < 0.001; ∗∗∗∗, *p* < 0.0001).

We used the TCGA database to examine the expression of BCL2L12 in CRC tumors and neighboring normal tissues. The results showed that the expression of BCL2L12 in CRC tissues was significantly higher than that in normal tissues (Figures 6(a) and 6(b)). Then, we evaluated the clinical value of BCL2L12 in CRC. The univariate analysis revealed that BCL2L12 was an independent factor of age, T stage, N stage, M stage, pathologic stage, and lymphatic invasion (Figure 6(c)). In addition, we evaluated the risk score of BCL2L12 in CRC patients and revealed that the high-risk group had a higher mortality rate, with the similar trend of hsa-miR-3937 (Figure 6(d)). In the next part, we further detected the differential expression genes (DEGs) related to BCL2L12 in CRC. CRC samples were split into BCL2L12^high^ and BCL2L12^low^ group based on BCL2L12 gene expression levels. We examined DEGs between BCL2L12^high^ and BCL2L12^low^ groups using |log2*FC*| > 2 and adjusted *p* < 0.05. The heatmap showed the top 50 of DEGs (Figure 7(a)). Then, we analyzed GSEA results of DEGs associated with BCL2L12 in CRC by TCGA database, enriching in EMT signature (Figure 7(b)).

**Figure 8:**
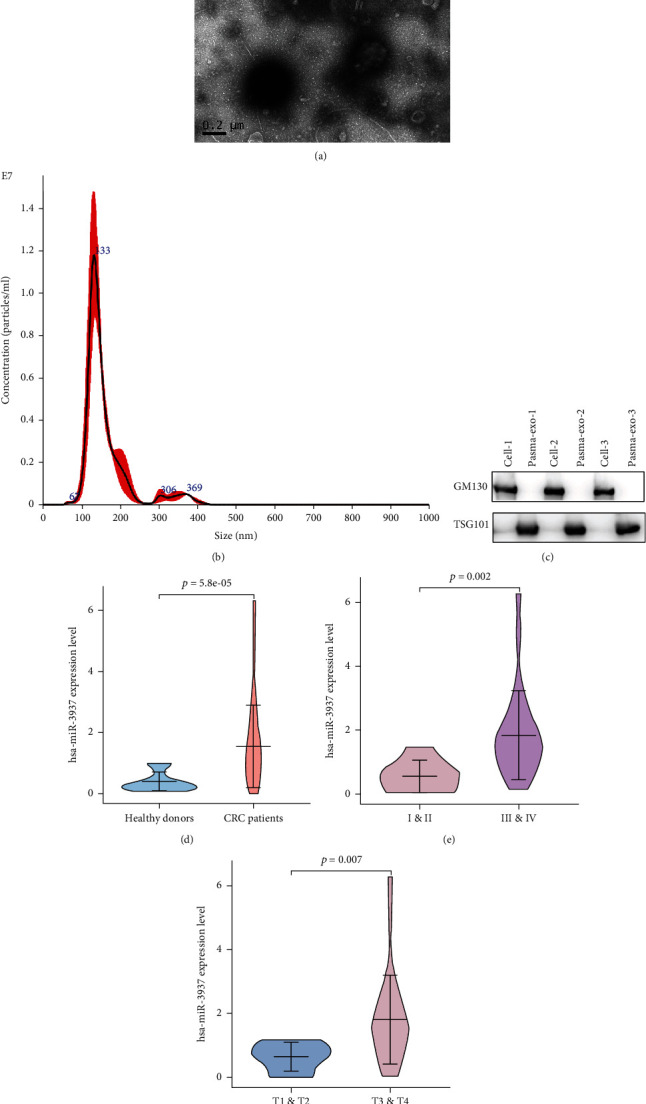
hsa-miR-3937 was upregulated in CRC plasma-derived exosomes. (a) TEM identifies the characteristics of plasma-derived exosomes. (b) NTA shows the distribution and size of exosomes. (c) Western blot was used the identify the markers of exosomes. (d) Exosomal hsa-miR-3937 expression levels in plasma were significantly upregulated in CRC patients when compared with healthy controls. (e) Exosomal hsa-miR-3937 expression levels were significantly upregulated in advanced (III and IV) CRC patients' plasma. (f) Exosomal hsa-miR-3937 expression levels were significantly upregulated in T3 and T4 CRC patients' plasma.

To learn more about BCL2L12, we utilized KEGG and GO enrichment studies. As Figures 8(c) and 8(b) shown, the BCL2L12 downstream function enriched in systemic lupus erythematosus, alcoholism, and so on. These results provided us the further research direction.

### 3.6. Plasma Exosomal hsa-miR-3937 as a Novel Biomarker of Colorectal Cancer

**Figure 9:**
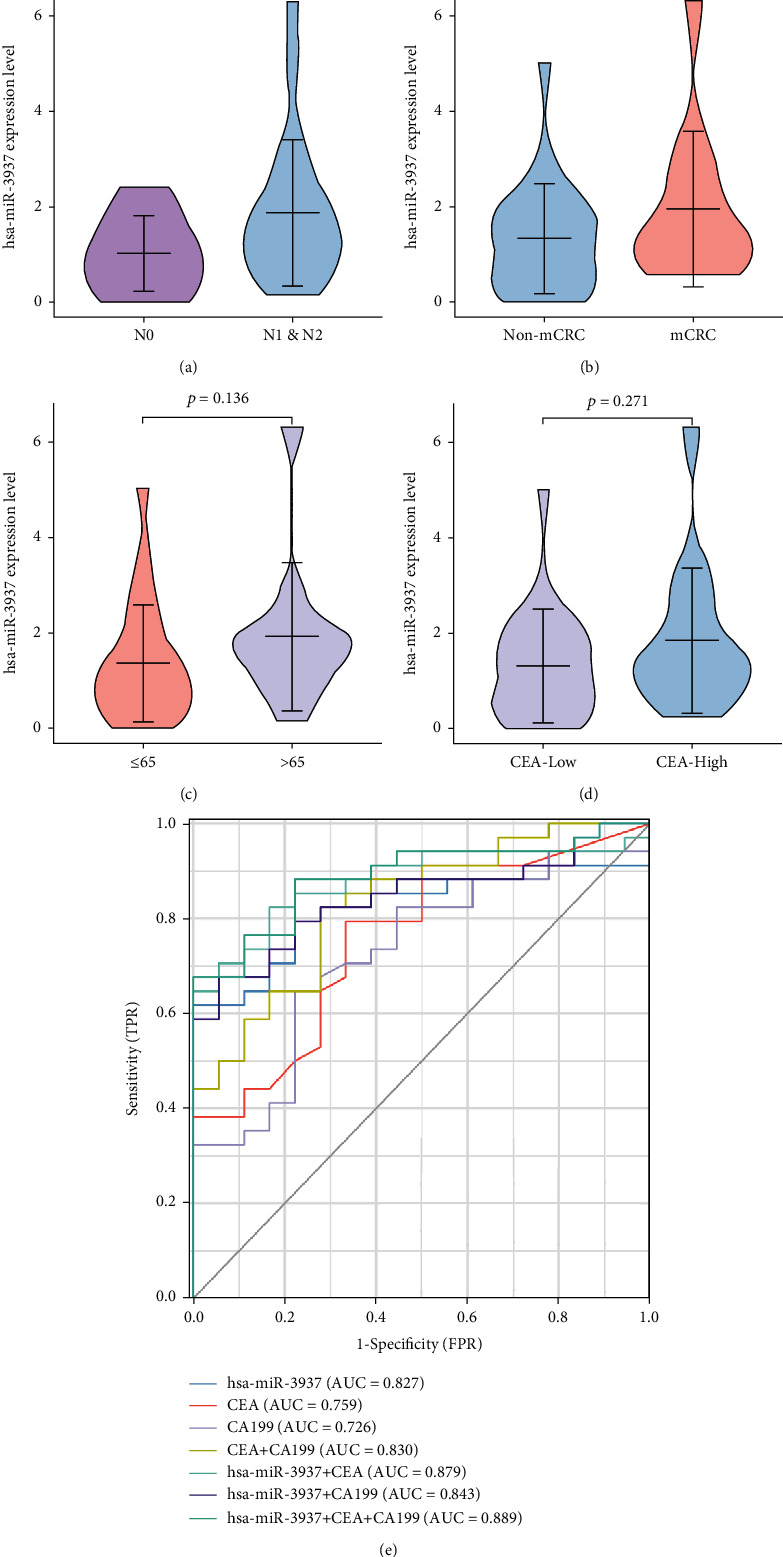
Correlations between plasma exosomal hsa-miR-3937 levels and CRC clinical parameters and diagnostic power of each marker for CRC. (a) No statistical differences were observed in plasma exosomal hsa-miR-3937 levels between stages N0 and N1&N2 patients. (b) No statistical differences were observed in plasma exosomal hsa-miR-3937 levels between non-mCRC and mCRC patients. (c and d) Plasma exosomal hsa-miR-3937 levels was not associated with age and CEA level of CRC patients. (e) The AUC of exosomal hsa-miR-3937 (0.827), CEA (0.759), and CA199 (0.726), and AUC for hsa-miR-3937 combined with CEA and CA199, hsa-miR-3937 combined with CEA, and hsa-miR-3937 combined with CA199 were 0.889, 0.879, and 0.843.

To further evaluate the clinical value of hsa-miR-3937 of CRC, we choose peripheral blood samples which were more accessible than tissues of patients. Firstly, we isolated the exosomes from 34 CRC patients' and 18 healthy donors' plasma samples and identified them, choosing the same methods as tissue-derived exosomes, including TEM, NTA, and western blot (Figures 8(a)–8(c)). The clinical characteristics are shown in Table 1. RT-qPCR was used to quantitatively assess hsa-miR-3937 expression levels in plasma samples. As the data shown, we detected that hsa-miR-3937 expression levels in CRC patients were significantly upregulated, compared with the healthy donors (*p* < 0.001) (Figure 8(d)). We also analyzed plasma exosomal hsa-miR-3937 expression levels in CRC patients at different clinical characteristics. From Figure 8(e), exosomal hsa-miR-3937 expression levels were higher in advanced CRC patients' plasma (*p* = 0.002). And the expression level of exosomal hsa-miR-3937 was associated with T stage (*p* = 0.007) (Figure 8(f)). However, there were no significant differences in plasma exosomal hsa-miR-3937 expression levels between N0 and N1 and N2 (*p* = 0.104) (Figure 9(a)). Moreover, there is no difference between metastatic CRC (mCRC) and nonmetastatic CRC (non-mCRC) as well as N stage (Figure 9(b)). The similar results happened to age and CEA levels (Figures 9(c) and 9(d)). In the last, we evaluated the predictive value of hsa-miR-3937 using ROC curve analysis. From Figure 9(e), the AUC (95% CI) for plasma exosomal hsa-miR-3937, CEA, and CA199 which were CRC diagnostic biomarkers were 0.827 (95% CI; 0.712-0.942), 0.759 (95% CI; 0.624-0.894) and 0.726 (95% CI; 0.584-0.894), respectively. And AUC for hsa-miR-3937 combined with CEA and CA199, hsa-miR-3937 combined with CEA, and hsa-miR-3937 combined with CA199 were 0.889 (95% CI; 0.801-0.977), 0.879 (95% CI; 0.785-0.974), and 0.843 (95% CI; 0.738-0.949). Analysis of these results, we concluded that these two or three indicators were combined, the diagnostic value was in more sensitivity and superior to CEA.

## 4. Discussion

Colorectal cancer was detected occasionally few decades ago. With about 850 000 fatalities each year, it is now the world's third most lethal malignancy [23]. The early detection of cancer has garnered significantly greater attention as a clinical problem that must be overcome [24]. Exosomes and their involvement in intercellular and intracellular communication have received increasing attention in recent years [20]. Exosomes are small extracellular vesicles secreted by a variety of different types of cells in the human body. DNA, RNA, proteins, and lipids comprise them. Cancer cells leak more exosomes than normal cells, making them potential candidates for cancer screening biomarkers in the future [22]. Recent years have seen an increase in interest in exosome-derived microRNA (miRNA). Their significance in the development of several forms of cancer, in particular, has been widely explored. Exosomal miRNAs (exomiRs) may function as either oncogenes or oncosuppressors. Because of their great stability and ease of detection in human fluids, they offer potential prognostic and diagnostic effectiveness in several forms of cancer. miR-155 has been found to generate drug resistance to paclitaxel and doxorubicin in breast cancer (BC) and to be an oncogenic signal [25]. In terms of prognosis, research found that increasing levels of exomiR-96 were associated with advanced tumor stage and grade, as well as lymph node metastasis in lung cancer patients, and that disease progression was controlled by exomiR-96 by targeting the tumor-suppressor LIM-domain-only protein 7 (LMO7) [26]. Increased plasma exosome levels of miR-1246 and miR-21 have been demonstrated to differentiate BC patients from healthy controls [27, 28]. These grants demonstrated the potential use of exosomal miRNA as cancer biomarkers.

In this work, we thoroughly and methodically assessed the involvement of hsa-miR-3937 in CRC. We chose and identified hsa-miR-3937, which was abundant in tumor-generated exosomes, based on earlier RNA sequencing data of exosomes obtained and extracted from seven matched specimens of tumor tissues and surrounding normal tissues of CRC patients. We performed the univariate Cox regression analysis to analyze the connection between has-miR-3937 expression and overall survival in two cohorts from the TCGA database to learn more about the effect of patients' has-miR-3937 expression in CRC. In addition, we looked at the hsa-miR-3937 risk score in CRC patients and discovered that the high-risk group had a higher death rate. The has-miR-3937 was then investigated as an independent predictor of survival in CRC patients. Then, using the Transwell assay, we determined the function of hsa-miR-3937 in CRC cells. Silencing hsa-miR-3937 decreased CRC cell invasion and migration. Furthermore, the TUNEL test in the SW480 cell line demonstrated that there was no significant difference between the expression level of hsa-miR-3937 and apoptosis, which is consistent with flow cytometry results. To gain insight into the mechanism by which hsa-miR-3937 operates as an oncogenic miRNA in CRC cells, we used TargetScan to estimate the binding location of hsa-miR-3937 on the target gene BCL2L12. Furthermore, in the SW480 cell line, downregulating BCL2L12 inhibited cell apoptosis, resulting in a lower number of siRNA-BCL2L12 (14.43%) than in the control group (19.01%). Furthermore, we used the TCGA database to investigate the expression levels of BCL2L12 in CRC tissues and neighboring normal tissues, and we hypothesized the probable downstream molecular route. The downstream function of BCL2L12 is enriched in systemic lupus erythematosus, alcoholism, and other diseases. These findings pointed us in the right path for further investigation.

Peripheral blood samples were more accessible than tissues of patients. To further evaluate the clinical value of hsa-miR-3937 of CRC and realize the feasibility of clinical diagnosis, we isolated the exosomes from 34 CRC patients' and 18 healthy donors' plasma samples. The data showed that hsa-miR-3937 expression levels in CRC patients were significantly upregulated, compared with the healthy donors (*p* < 0.001). Exosomal hsa-miR-3937 expression levels were higher in advanced CRC (III and IV) patients' plasma (*p* = 0.002). And the expression level of exosomal hsa-miR-3937 was associated with T stage (*p* = 0.007). However, there were no significant differences in plasma exosomal hsa-miR-3937 expression levels between N0 and N1 and N2 (*p* = 0.104). Moreover, there is no difference between metastatic CRC (mCRC) and nonmetastatic CRC (non-mCRC) as well as N stage. The similar results happened to age and CEA levels. The AUC (95% CI) for plasma exosomal hsa-miR-3937 was higher than CEA and CA199. Interestingly, AUC for hsa-miR-3937 combined with CEA and CA199 is superior to other combinations. All the results indicated that exosomal hsa-miR-3937 may be a novel auxiliary diagnostic marker for CRC diagnosis.

## 5. Perspectives and Future Directions

The lack of precise diagnosis technology has escalated the cost of CRC. To reduce the risk of death associated with this illness, more effective detection methods for this malignancy are required. Exosomes include RNAs and proteins that are differentially expressed in colorectal cancer (CRC). These chemicals may alter colorectal cancer oncogenesis, metastasis, chemoresistance, and recurrence, making them potential treatment possibilities. They are more sensitive and specific than certain traditional methods, contain a variety of bioactive molecules with less serum interference, and have high stability, and their contents are not degraded in the extracellular environment and can be extracted noninvasively from a variety of body fluids. Thus, exosomes may be used as novel tumor indicators.

Exosomes in the circulation must also be identified. Most cells can release exosomes into the bloodstream, and their apparent variability suggests that their contents and surface proteins may vary. It will need further study to differentiate between normal and cancerous exosomes in the blood. In other words, blood exosomes may be classified as blood cells. Finally, universal biomarkers for CRC patients have yet to be discovered. More comparative research with larger sample numbers is required to properly grasp the problem. Exosomes as a whole may be a source of biomarkers for colorectal cancer that may be detected noninvasively in patients' bodily fluids in the not too distant future.

## Figures and Tables

**Table 1:** Baseline analysis of clinical cases.

	Validation cohort
Total number	34
Age (years)	
Median (min~max)	64 (22~79)
Gender	
Female	13
Male	21
Pathologic stage	
I and II	8
III and IV	26
TNM staging	
T1 and T2	8
T3 and T4	26
N0	13
N1 and N2	21
M0	22
M1	12
CEA	
Low	19
High	15

## Data Availability

The datasets supporting the conclusions of this article are included within the article.
